# MAPT mutation-induced behavioral variant frontotemporal dementia in an Asian patient: a multimodal biomarker case report resolving diagnostic challenges with Alzheimer’s disease

**DOI:** 10.3389/fgene.2025.1645068

**Published:** 2025-11-18

**Authors:** Yan Zhang, Siwei Chen, Guiying Yan, Zhifei Zhang, Amina, Ting Wang, Shuang Wang, Chen Zhang, Yong’an Sun

**Affiliations:** 1 Department of Neurology, Peking University First Hospital, Beijing, China; 2 Academy of Mathematics and Systems Science, Chinese Academy of Sciences, Beijing, China

**Keywords:** frontotemporal dementia, MAPT mutation, biomarkers, cross-culturaldiagnosis, NMDA receptor antagonist

## Abstract

**Background:**

The clinical phenotypic overlap between frontotemporal dementia (FTD) and Alzheimer’s disease (AD) frequently leads to misdiagnosis, while biomarkers (e.g., Aβ-PET) and genetic testing provide critical differential diagnostic evidence. Although MAPT gene mutations represent common genetic etiologies of FTD, their occurrence in Asian populations remains underreported. Specifically, FTD caused by the MAPT IVS10 + 16C>T mutation shows limited documentation in Asian populations, with its phenotypic heterogeneity and treatment responses remaining poorly characterized.

**Methods:**

We present a case of FTD manifesting progressive memory decline, compulsive behaviors, and apathy. MRI revealed bilateral frontoparietotemporal atrophy with prominent medial temporal lobe and hippocampal involvement, initially misdiagnosed as AD. Subsequent Aβ-PET negativity emerged as a pivotal diagnostic turning point, and identification of a heterozygous MAPT mutation (IVS10 + 16C>T) confirmed behavioral variant FTD (bvFTD) diagnosis. Partial improvement in compulsive behaviors and verbal fluency was observed following memantine treatment. A literature review summarizes clinical characteristics of FTD associated with IVS10 + 16C>T mutations.

**Results:**

Comprehensive neuropsychological assessment, cranial MRI, and negative Aβ-PET excluded AD pathology. Genetic confirmation of MAPT IVS10 + 16C>T mutation established bvFTD diagnosis. Three-month memantine treatment reduced compulsive behaviors without cognitive improvement. Literature analysis indicates this mutation’s rarity in Asian populations, typically presenting with behavioral abnormalities frequently misdiagnosed as AD.

**Conclusion:**

This study rectified misdiagnosis of MAPT IVS10 + 16C>T-associated bvFTD through multimodal diagnostics, emphasizing the synergistic value of genetic testing and neuroimaging. Memantine’s partial behavioral symptom alleviation suggests potential mutation-specific therapeutic efficacy requiring further validation. Future directions should optimize diagnostic protocols (e.g., cost-effective genetic screening) and address barriers to early diagnosis in Asian populations.

## Highlights

- Report the association between MAPT IVS10 + 16C>T mutation and bvFTD phenotype in Asian populations.
- Reveal the clinical value of imaging-genetic combined diagnosis in Aβ-PET negative contexts.
- Propose the therapeutic hypothesis of memantine efficacy for MAPT mutation-related FTD.

## 1 Introduction

Frontotemporal dementia (FTD) accounts for 20% of early-onset dementia cases, characterized by core features of behavioral abnormalities and language impairment (Author Anonymous, 1994), The pathological mechanisms are frequently associated with mutations in MAPT, GRN, or C9orf72 genes, with other rare pathogenic genes identified in less than 5% of cases (Greaves and Rohrer, 2019). Genetic studies of Chinese FTD populations have revealed genotype-phenotype correlations: MAPT mutations with frontotemporal dementia and parkinsonism linked to chromosome 17 (FTDP-17), GRN mutations with frontotemporal lobar degeneration with TDP-43 proteinopathy (FTLD-TDP), C9orf72/CHCHD10/TBK1 mutations with amyotrophic lateral sclerosis (ALS)-FTD spectrum disorders, and VCP mutations with inclusion body myopathy associated with Paget disease of bone and frontotemporal dementia (IBMPFD) (Jiang et al., 2021). Notably, genetic mutations do not consistently predict clinical phenotypes, as different mutations within the same gene may manifest distinct clinical presentations. In China, MAPT gene mutations represent a predominant pathogenic factor for FTD, underscoring the clinical significance of understanding genotype-phenotype correlations for diagnosis and management (Jiang et al., 2021). The MAPT gene exhibits over 80 identified mutations associated with various tauopathies, yet no unified mechanism explains tau mutation effects, resulting in phenotypic heterogeneity and therapeutic development challenges (De wit and Ghosh, 2016). Current FTD management lacks disease-modifying therapies. Memantine, an FDA-approved N-methyl-D-aspartate (NMDA) receptor antagonist for Alzheimer’s disease (AD), theoretically mitigates glutamate-mediated neurotoxicity through non-competitive NMDA receptor blockade. Although FTD neuropathology differs from AD, memantine’s neuroprotective properties may offer therapeutic benefits. Supporting this hypothesis, memantine’s efficacy in managing behavioral symptoms of AD has prompted investigation for FTD treatment (Xu et al., 2024). However, two independent double-blind, placebo-controlled trials demonstrated no significant improvement between groups, with potential cognitive deterioration observed in memantine-treated patients (Vercelletto et al., 2011; Boxer et al., 2013).

We report a case initially misdiagnosed as Alzheimer’s disease (AD) that was ultimately confirmed as behavioral variant frontotemporal dementia (bvFTD) through multimodal diagnostic approaches (clinical, imaging, and genetic). Through literature review, this study further elucidates the association between MAPT IVS10 + 16C>T mutation and bvFTD phenotype in Asian populations, providing new evidence for cross-cultural genetic research and personalized therapeutic strategies. We propose a hypothesis regarding the potential efficacy of memantine in MAPT mutation-related FTD.

## 2 Case report

A 60-year-old female with a 5-year disease course presented with progressive cognitive decline and behavioral abnormalities. Initial symptoms manifested as short-term memory impairment, characterized by frequent forgetfulness of daily activities (e.g., misplacing bicycles and repeatedly reporting to police) and repetitive questioning about resolved issues (e.g., dinner arrangements). Progressive behavioral changes emerged during disease progression: marked compulsive behaviors (strict regulation of children’s morning routines with emotional outbursts for non-compliance; hoarding irrelevant items and coercing children to accept them), emotional blunting (lack of spontaneous communication, mechanical responses to family members), hyperorality with carbohydrate craving, and disinhibition (socially inappropriate behaviors including unprompted lip-smacking and excessive environmental cleanliness criticism). Initial brain MRI at another institution revealed symmetric temporal lobe and hippocampal atrophy, leading to AD diagnosis based on memory impairment as the initial presentation. The patient was subsequently referred to our institution for further evaluation.

## 3 Past medical history and family background

The patient had a history of insulin-dependent type 2 diabetes mellitus and underwent minimally invasive surgery for cervical spondylosis 5 years prior. No family history of dementia was reported. She completed undergraduate education and had been retired for 5 years.

## 4 Differential diagnostic considerations at initial presentation

At symptom onset, the differential diagnosis primarily included Alzheimer’s disease (AD), given the prominence of memory complaints. However, the early age of onset (55 years) and the subsequent emergence of distinct behavioral features (compulsivity, disinhibition, hyperorality) raised suspicions of alternative etiologies. Key differentials considered were: Alzheimer’s Disease (AD): Supported by the initial memory impairment and medial temporal lobe atrophy on MRI. Behavioral Variant Frontotemporal Dementia (bvFTD): Suggested by the progressive behavioral changes, including compulsivity, disinhibition, and hyperorality, which are core diagnostic features. Other Neurodegenerative Disorders: Corticobasal degeneration (CBD) or progressive supranuclear palsy (PSP) were considered less likely in the absence of prominent motor signs initially. This diagnostic ambiguity underscores the challenge of distinguishing AD from bvFTD based on early clinical features alone, particularly when memory deficits are prominent.

## 5 Diagnostic workup and management

**FIGURE 1:**
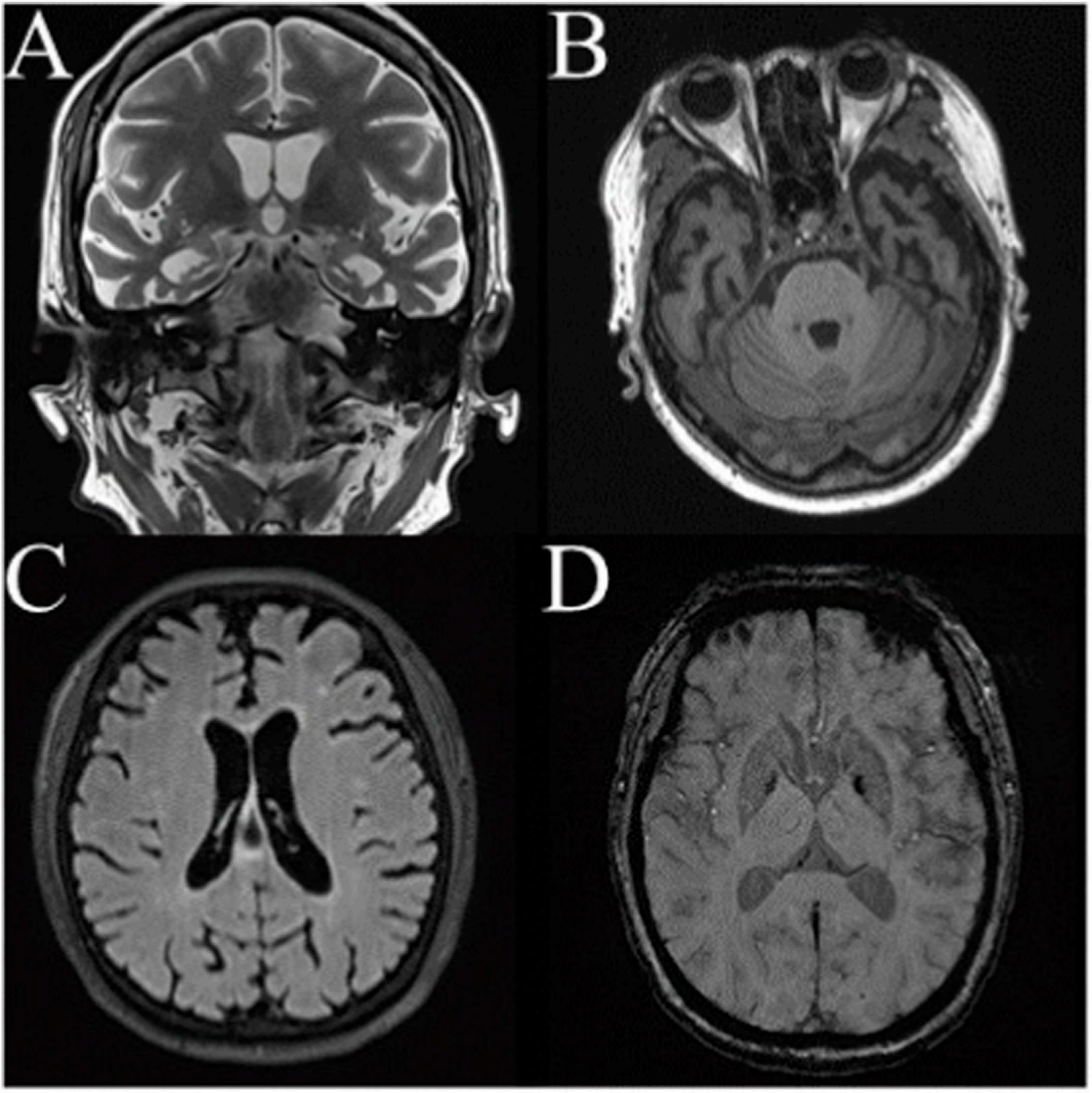
**(A)** Coronal hippocampal imaging reveals bilateral hippocampal atrophy (MTA grade 4). **(B)** Marked bilateral temporal pole atrophy is evident. **(C)** Scattered small ischemic foci are observed in both hemispheres, with no extensive white matter lesions or strategic infarcts. **(D)** Microbleed sequences show a single microbleed in the left occipital lobe, without evidence of multiple lobar microbleeds or cortical superficial siderosis.

Neuropsychological assessment revealed significant cognitive decline (MMSE 17/30, MoCA 10/30) with predominant compulsive and disinhibited behaviors (NPI 33). Multimodal MRI demonstrated prominent bilateral atrophy in frontal, parietal, and temporal lobes (including mesial temporal regions and temporal poles), bilateral hippocampal atrophy (MTA grade 4, Figure 1). Given the initial presentation of memory impairment requiring AD differentiation, Aβ-PET imaging was performed and showed no significant radiotracer uptake, effectively excluding AD pathology. This finding was a critical turning point, redirecting the diagnostic focus towards non-AD dementias, specifically FTD. Cerebellar uptake remained normal, with notable supratentorial ventricular enlargement and widened cerebral sulci. Blood biomarkers revealed elevated p-tau181 (8.344 pg/mL, normal <5.0) with normal Aβ42, Aβ42/40 ratio, p-tau217, GFAP, and nfl levels. Whole-exome sequencing (WES) identified MAPT IVS10 + 16C>T heterozygous mutation (a previously reported pathogenic variant for FTD) and APOE ε2/ε3 genotype (ApoE2), confirming the diagnosis of bvFTD.

**FIGURE 2:**
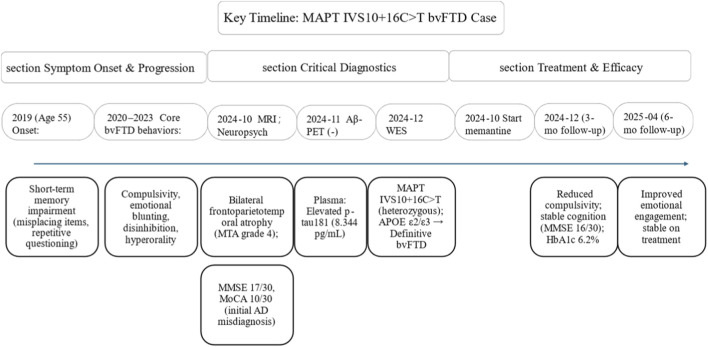
Timeline of key clinical events, diagnostics, and treatment for the MAPT IVS10 + 16C>T-associated bvFTD patient, including symptom progression (2019–2023), stepwise diagnosis (2024: MRI, Aβ-PET, plasma biomarkers, WES), and 3/6-month treatment follow-up.

Therapeutic intervention including memantine (20 mg/d) and blood glucose management and family care demonstrated partial clinical improvement at 3-month follow-up: family reported reduced compulsive behaviors and improved verbal responsiveness. Persistent hyperorality and sugar craving manifested as candy hoarding. MMSE and MoCA scores remained stable. Combined diabetes management achieved HbA1c reduction from 10.2% to 6.2%. At 6-month evaluation, modest improvement in emotional blunting was observed, with increased spontaneous text/emoji communication in family chat groups. The patient currently maintains stable clinical status with ongoing monitoring of cognitive-behavioral progression. Figure 2 shows the key timeline of events for the patient.

## 6 Discussion

### 6.1 Paradoxical biomarkers: the diagnostic dilemma between AD and FTD

Frontotemporal dementia (FTD), a clinically, pathologically, and genetically heterogeneous neurodegenerative disorder, is frequently misdiagnosed as Alzheimer’s disease (AD), the most prevalent etiology of dementia. Clinicians typically prioritize an AD diagnosis when imaging and clinical manifestations align with AD progression. In this case, the patient initially presented with pronounced memory impairment, recent memory decline, and symmetrical atrophy in bilateral frontoparietotemporal regions alongside severe hippocampal atrophy, prompting initial consideration of AD. However, subsequent emergence of hallmark behavioral abnormalities—compulsive behaviors, apathy, disinhibition, and hyperorality—shifted diagnostic suspicion toward FTD. The diagnostic challenge arises from overlapping clinical features between AD and FTD, particularly in advanced stages, coupled with the lack of specific, widely accepted biomarkers for FTD subtypes (Swift et al., 2021). Although MRI revealed temporal lobe atrophy (a typical AD feature), Aβ-PET negativity served as a pivotal biomarker that definitively excluded AD pathology, underscoring the pivotal role of biomarkers in differential diagnosis. Notably, while some clinical AD cases exhibit Aβ-PET negativity, and certain FTD patients may display temporal atrophy due to comorbid tau pathology, genetic testing remains critical for definitive diagnosis. Here, the identification of a MAPT mutation directly implicated tauopathy, supporting classification as a “non-AD dementia” (Jack et al., 2024). Intriguingly, elevated plasma p-tau181 levels were observed in this patient, while AD-associated biomarkers (p-tau217, Aβ42, and Aβ42/40 ratios) remained within normal ranges, further negating an AD diagnosis. Prior studies indicate that plasma p-tau181 levels in AD patients are approximately 3.5-fold higher than in controls, correlating with severity of gray matter atrophy in AD-affected regions, and are considered relatively specific to AD. However, recent research by Professor Lu Shen’s team demonstrated statistically significant p-tau181 elevation in both behavioral variant FTD (bvFTD) and FTD with parkinsonism (FTD-P) compared to controls (Sayegh and Knight, 2013). Suggesting shared clinicopathological mechanisms between FTD and AD. This finding aligns with the overlapping clinical features observed in this case (Thijssen et al., 2020).

### 6.2 MAPT mutations in Asian populations: genetic heterogeneity and phenotypic specificity

The MAPT IVS10 + 16C>T mutation is exceptionally rare in Asian populations, exhibiting marked genetic heterogeneity and cross-cultural phenotypic variability. Previous reports describe misdiagnosis of mutation carriers as early-onset AD, exemplified by Doran et al. (2016), where four family members initially presented with AD-like memory deficits; diagnoses were revised to FTD only after neuropathological confirmation of tauopathy and genetic identification of MAPT mutations. Conversely, other family members manifested behavioral disturbances (motor restlessness, dietary changes) and executive dysfunction, highlighting phenotypic diversity (Horimoto et al., 2024). Table 1 summarizes clinical features associated with this mutation (adapted from Larner et al.), demonstrating its heterogeneous manifestations: the same mutation may cause bvFTD, AD-like phenotypes, parkinsonism, or progressive supranuclear palsy (PSP)-like symptoms, often initially misdiagnosed as early-onset AD. Affected individuals exhibit broad age-of-onset variability (37–62 years), male predominance, early behavioral changes (disinhibition, compulsivity, hyperphagia, stereotypic movements), and frequent progression to parkinsonism (30%–50% of cases). Some cases present with atypical AD or PSP-like features (e.g., memory impairment, supranuclear gaze palsy), yet all share tauopathy as the neuropathological basis. This heterogeneity may stem from modifier genes, environmental factors, or unidentified mechanisms, emphasizing the need for heightened suspicion of FTD in early-onset dementia with behavioral or motor symptoms. Family history (even if labeled as “AD” or “Parkinson’s disease”) and genetic testing are critical to avoid misdiagnosis (Horimoto et al., 2024). SuStaIn analysis further links the IVS10 + 16C>T mutation to focal medial temporal atrophy, overlapping with AD-associated temporal atrophy and increasing misdiagnosis risk (Young et al., 2021), However, Aβ-PET negativity and genetic confirmation in this case unequivocally supported FTD, reinforcing the necessity of multimodal assessment combining neuroimaging (e.g., medial temporal atrophy), biomarkers (Aβ-PET, plasma AD markers), and genetic analysis. MAPT mutation-specific atrophy patterns further inform diagnosis: IVS10 + 16C>T and R406W mutations correlate with temporal atrophy and memory deficits, whereas P301L mutations associate with frontotemporal atrophy and executive dysfunction (Young et al., 2021). Longitudinal follow-up is warranted to monitor potential parkinsonian or PSP-like manifestations in this patient. This patient experienced a 5-year diagnostic delay. The aforementioned literature does not provide specific mean or median figures for the diagnostic delay in IVS10 + 16C>T mutation carriers; however, the studies emphasize that clinical heterogeneity is a major cause of diagnostic difficulty. For instance, the case described by Morris et al. (2003) presented with a PSP-like phenotype, which could have led to initial misdiagnosis as other parkinsonian or movement disorders. Cases reported by Doran et al. (2007) exhibited AD-like symptoms, which similarly would prolong the time to definitive diagnosis. Based on current literature, there appear to be no clear statistical data indicating the specific duration of diagnostic delay for the IVS10 + 16C>T mutation. Many studies highlight the diagnostic challenges rather than quantifying the delay (Larner, 2009a). For example, the “Annual of the Pathological Autopsy Cases in Japan” reported that among 204 pathologically confirmed frontotemporal lobar degeneration (FTLD) cases, 154 were clinically overlooked, including 13 misdiagnosed as Alzheimer’s disease, indicating that misdiagnosis and delayed diagnosis are widespread (Horimoto et al., 2024).

**TABLE 1:** Case reports of IVS10 + 16C>T mutation carriers (Horimoto et al., 2024).

Reference	Sample size (Male:Female)	Age (years)	Clinical manifestations (disease onset and follow-up)
Pickering-Brown (Pickering-Brown et al., 2002)	9M:4F	46–62	Universal disinhibition, hyperorality, emotional blunting. Parkinsonism in 4 cases
Janssen (Janssen et al., 2002)	24M:14F	37–59	Behavioral/personality changes, compulsions, and hyperorality were observed. Some cases exhibited disinhibition (23 cases), with parkinsonism developing in 12 out of 23 cases
Morris (Morris et al., 2003)	1M	40	Sporadic young-onset progressive supranuclear palsy (PSP) phenotype
Stanford (Stanford et al., 2004)	1	54–57	Frontotemporal dementia with parkinsonism (FTDP) presentation
Doran (Doran et al., 2007)	5 M	39–50	Initial “AD” diagnosis, later developed behavioral variant FTD (bvFTD)
Larner (Horimoto et al., 2024; Larner, 2008; Larner, 2009b)	3 M	45–56	Initial “AD” diagnosis, later developed behavioral changes or PSP features. Family history of “early-onset AD” or FTD.

### 6.3 Controversial efficacy of memantine: Mechanistic hypotheses and subtype specificity

Currently, there are no effective treatments for frontotemporal dementia (FTD), largely due to incomplete understanding of its pathogenesis. Memantine, which improves cognitive symptoms in Alzheimer’s disease (AD) by blocking NMDA receptor overactivation, exhibits conflicting evidence for efficacy in FTD. The symptomatic improvement observed in this patient following memantine administration may be attributed to two mechanisms: Synaptic Plasticity Modulation: MAPT mutations induce glutamatergic neuronal hyperexcitability in the frontal lobe, and memantine may mitigate synaptic toxicity. However, this observation is based on a single case and requires further validation through larger, rigorously controlled clinical trials to confirm its generalizability and mechanistic certainty. Behavioral Symptom Targeting: Compulsive behaviors are linked to frontal-striatal circuit dysfunction, with animal models demonstrating memantine’s ability to reduce repetitive stereotypic behaviors (Mcshane et al., 2019). For the improvement in behavioral symptoms observed in this patient, this remains a preliminary observation and cannot be considered conclusive evidence of memantine’s efficacy. However, sustained monitoring of behavioral and cognitive changes remains essential. Current pharmacological research for FTD focuses on alleviating behavioral symptoms and exploring disease mechanisms. Selective serotonin reuptake inhibitors (SSRIs), such as paroxetine (20 mg/day improves behavioral symptoms but higher doses may impair cognition), citalopram (30 mg/day significantly reduces disinhibition and depression), and sertraline (ameliorates stereotypic behaviors), modulate serotonergic networks to partially address disinhibition, impulsivity, and dietary dysregulation in FTD. However, studies are limited by dose-dependent effects and small sample sizes. Trazodone reduces agitation/aggression and aids sleep, while second-generation antipsychotics (e.g., quetiapine, olanzapine) are commonly used for agitation/psychosis but constrained by extrapyramidal side effects and FDA black-box warnings (stroke risk). Anticonvulsants (topiramate, valproate) and lithium carbonate show anecdotal benefits for hyperphagia or disinhibition but lack robust evidence and carry metabolic/cognitive risks. Dopaminergic stimulants (methylphenidate) and intranasal oxytocin demonstrate potential for improving apathy, decision-making, and emotion recognition, though most trials are small-scale or short-term. Traditional agents like memantine and cholinesterase inhibitors are not recommended due to insufficient efficacy. Emerging research prioritizes gene-targeted therapies (e.g., tau reduction, AAV-mediated GRN delivery, targeting C9orf72 repeats) and biomarker development (neurofilament light chain, MRI monitoring) to modify molecular pathology and delay progression, though most remain in clinical trials (Guimet et al., 2022), Clinically, risk-benefit assessment and personalized treatment selection are critical for behavioral management. Beyond pharmacological intervention, comprehensive management strategies, such as controlling vascular risk factors, optimizing the management of comorbidities (e.g., diabetes in this case), and strengthening family care and support, may also contribute to stabilizing the patient’s overall condition and improving long-term outcomes.

### 6.4 Dementia diagnosis in Asia: cultural perceptions and healthcare delays

In Asian, the diagnosis of dementia, particularly frontotemporal dementia (FTD), faces significant challenges due to various cultural perceptions and healthcare resource limitations, leading to increased risks of diagnostic delays and misdiagnosis. One study notes that in young-onset dementia, the average time from symptom onset to initial assessment is 4–5 years, while the period from initial assessment to final diagnosis take several years; for example, the diagnostic delay for behavioral variant frontotemporal dementia (bvFTD) may be as long as 4.2 years (Xu et al., 2024). In many Asian cultures, abnormal behaviors are often attributed to “personality issues” or normal aging processes rather than underlying pathological changes (Sayegh and Knight, 2013). For instance, among dementia patients in China, the average time from symptom onset to first seeking medical diagnosis is 27 months, with the longest delay reaching 5 years, indicating substantial delays in seeking medical help (Zhao et al., 2016). Such issues also exist in other countries. A Dutch study found that the average time from symptom onset to diagnosis for early-onset dementia is 4.4 years, significantly longer than the 2.8 years for late-onset dementia. The study also revealed that frontotemporal dementia exhibits a longer diagnostic delay in both age groups, which may stem from its clinical manifestations—primarily behavioral and personality abnormalities—often being misinterpreted as psychological, mental, or interpersonal issues rather than neurological disorders. To address this situation, there is an urgent need to develop culturally adapted screening tools, promote low-cost biomarkers (such as plasma p-tau217) and genetic testing, and enhance doctor-patient communication and public education to reduce diagnostic delays and achieve more timely intervention and management (Loi et al., 2021).

## 7 Conclusion

This case highlights the pivotal role of biomarkers and genetic testing in differentiating atypical dementia phenotypes while providing novel evidence for MAPT mutation-associated phenotypic diversity in Asian populations. Whether memantine’s efficacy correlates with specific pathological subtypes requires further investigation. Future studies should explore precision therapeutic strategies for MAPT mutation-related FTD.

## 8 Innovative highlights

1. First report of the MAPT c.2091 + 16C>T mutation in Asia: This finding enriches genetic data for East Asian populations, where this mutation is exceptionally rare.
2. Hypothesis on therapeutic mechanisms: Linking memantine efficacy to specific MAPT mutation subtypes provides a theoretical foundation for future clinical trial design.
3. Cross-cultural healthcare analysis: Proposes optimized early dementia screening strategies tailored to sociocultural contexts in Asian communities.

## Data Availability

The original contributions presented in the study are included in the article/Supplementary Material, further inquiries can be directed to the corresponding author.
