# Correction: MAPT mutation-induced behavioral variant frontotemporal dementia in an Asian patient: a multimodal biomarker case report resolving diagnostic challenges with Alzheimer’s disease

**DOI:** 10.3389/fgene.2025.1749030

**Published:** 2025-12-01

**Authors:** Yan Zhang, Siwei Chen, Guiying Yan, Zhifei Zhang, Amina, Ting Wang, Shuang Wang, Chen Zhang, Yong’an Sun

**Affiliations:** 1 Department of Neurology, Peking University First Hospital, Beijing, China; 2 Academy of Mathematics and Systems Science, Chinese Academy of Sciences, Beijing, China

**Keywords:** frontotemporal dementia, MAPT mutation, biomarkers, cross-culturaldiagnosis, NMDA receptor antagonist

In the published article, the **Acknowledgments** section was omitted. The Acknowledgement section appears below.

The authors are thankful to the patient and her family for allowing us to share this case. We would like to express our gratitude to Dr. Meng Yu for referring the patient and to the patient’s family for their active cooperation during the diagnosis and treatment process, which significantly facilitated the smooth progression of the clinical workflow.

The original article has been updated.

