# Supplementary figures and images for: MAPT mutation-induced behavioral variant frontotemporal dementia in an Asian patient: a multimodal biomarker case report resolving diagnostic challenges with Alzheimer’s disease
Source: Front Genet. 2025 Nov 18;16:1645068. doi: 10.3389/fgene.2025.1645068 (PMC12702606; doi:10.3389/fgene.2025.1645068)

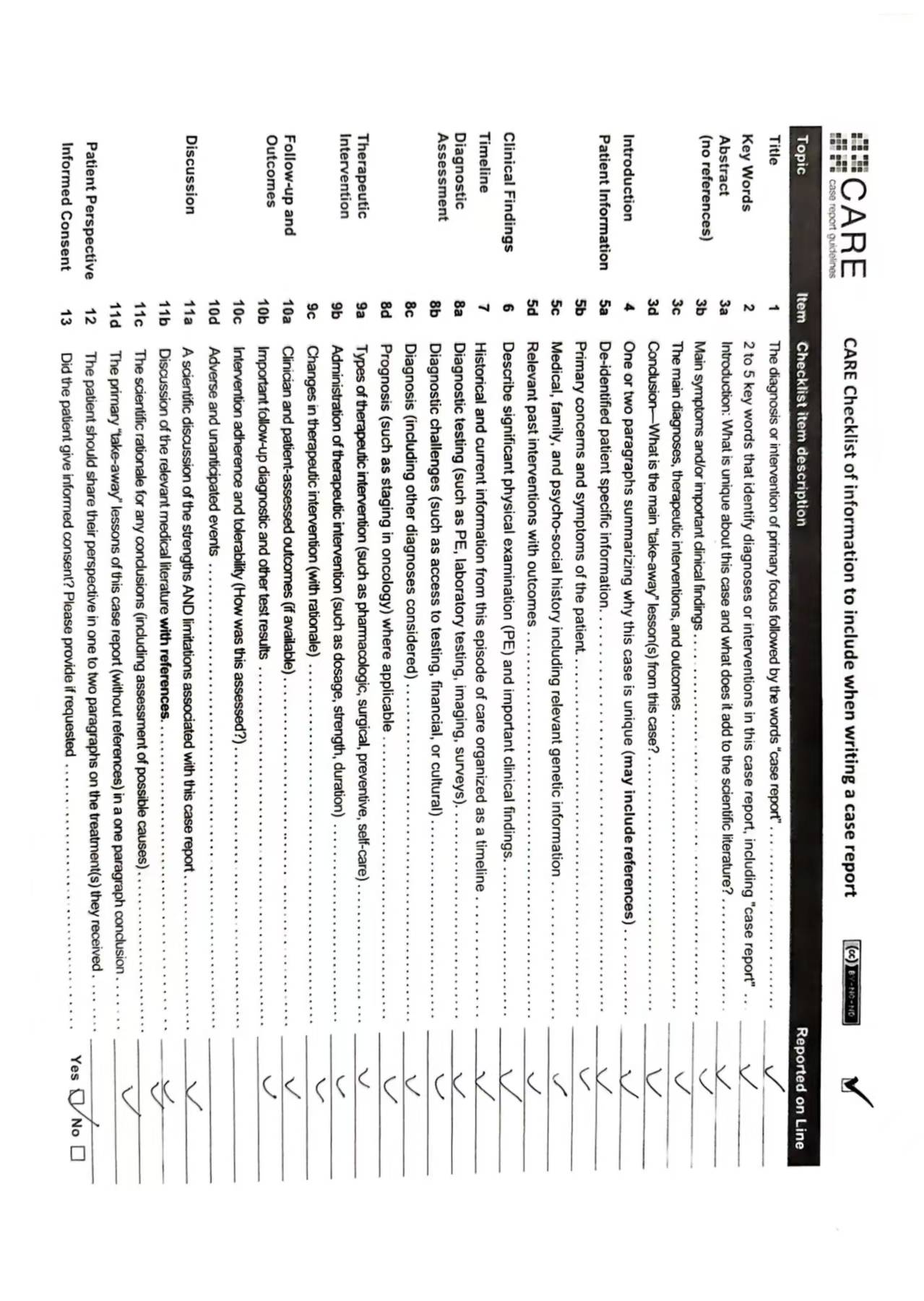

Supplement: Supplementary file 1 [file Table1.docx]
